# Relative Effects of Sensory Modalities and Importance of Fatty Acid Sensitivity on Fat Perception in a Real Food Model

**DOI:** 10.1007/s12078-016-9211-5

**Published:** 2016-07-11

**Authors:** Xirui Zhou, Yuchi Shen, Jane K Parker, Orla B Kennedy, Lisa Methven

**Affiliations:** 1 Sensory Centre, Department of Food and Nutritional Sciences, The University of Reading, PO Box 226, Whiteknights, Reading, RG6 6AP UK; 2 Flavour Centre, Department of Food and Nutritional Sciences, University of Reading, Reading, UK; 3 Hugh Sinclair Human Nutrition Unit, Department of Food and Nutritional Sciences, University of Reading, Reading, UK; 4 Department of Food and Nutritional Sciences, The University of Reading, PO Box 226, Whiteknights, Reading, RG6 6AP UK

**Keywords:** Fat perception, Taste, Fatty acid sensitivity, Detection threshold, Fat intake

## Abstract

**Introduction:**

Fat can be perceived through mouthfeel, odour and taste, but the influence of these modalities on fat perception remains undefined. Fatty acids are stimuli and individual sensitivity to fatty acids varies. Studies show association between fatty acid sensitivity, dietary intake and BMI, but results are conflicting. Therefore, this study examined this association, and the effect of modalities on fat perception.

**Methods:**

Two sub-studies were conducted. In study 1 (*n* = 46), fat intensity was assessed by milk/cream mixtures varying by five fat levels. Fat intensity was rated under four conditions: mouthfeel odour-masked, mouthfeel-masked, odour masked and with no masking. Mouthfeel masking was achieved using thickener and paraffin, odour masking using nose-clips. Fatty acid sensitivity was measured by 3-AFC staircase method using milk containing oleic acid (0.31–31.4 mM). In study 2 (*n* = 51), more fat levels were added into the intensity rating. A 2-AFC discrimination test was used to confirm whether fat levels could be distinguished. In the sensitivity test, a wider range of oleic acid was included.

**Results:**

Fat intensity was rated higher without nose clips (*p* < 0.0001), implying that odour increased fat perception. Mouthfeel-masked samples were rated higher, showing that increased viscosity and lubricity enhanced fat perception (*p* < 0.0001). Participants could distinguish fat levels based on “taste” in rating tests and 2-AFC tests. Participants were divided into high-/medium-/low-sensitivity groups. No significant difference was found in fat intensity between groups; however, the high-sensitivity group discriminated more fat levels. No association between sensitivity groups, nutrient intake or BMI was found.

**Conclusion:**

Mouthfeel and odour can enhance fat perception. Fat level can be discriminated based on taste.

**Electronic supplementary material:**

The online version of this article (doi:10.1007/s12078-016-9211-5) contains supplementary material, which is available to authorized users.

## Introduction

Obesity results from an energy imbalance and, as fat is the most energy-dense macronutrient in the diet, excess fat consumption coupled with low vegetable/fruit and high sweet/carbohydrate intake could increase the risk of developing obesity (McCrory et al. 1999). Modification of high fat foods by using fat replacers, such as cellulose and starch, has become a popular product strategy taken by food industries; however, such substitution of fat does not recreate the full sensory quality of fat in food (Gibis et al. 2015; Zahn et al. 2010). Traditionally, fat was considered to be perceived only through mouthfeel and olfaction, but more recent evidence suggests that fat can also be perceived though taste (Chale-Rush et al. 2007a; Keast and Costanzo 2015; Mattes 2009; Pepino et al. 2012; Running et al. 2015; Stewart et al. 2010; Tucker and Mattes 2012). Therefore, understanding the effects of taste, mouthfeel and odour on oral fat perception might be valuable for the development of sensorially improved low-fat foods. However, research in this area is limited.

Several studies indicate that free fatty acids are the main effective stimuli in fat taste perception (Chale-Rush et al. 2007b; Fukuwatari et al. 1997; Gilbertson et al. 1997; Gilbertson et al. 2005; Martin et al. 2011; Tsuruta et al. 1999), where researchers have shown that both humans and animals can detect the presence of fatty acid when mouthfeel and odour are both masked (Chale-Rush et al. 2007a; Chale-Rush et al. 2007b; Mattes 2011; Stewart et al. 2010; Stewart et al. 2011; Tucker et al. 2015). Individuals are reported to show different sensitivities to the taste of fatty acids (Stewart et al. 2010; Stewart et al. 2011; Chevrot et al. 2014; Martinez-Ruiz et al. 2014; Tucker et al. 2015), and some studies imply a potential association between fatty acid sensitivity and dietary intake (Stewart et al. 2010; Stewart et al. 2011; Martinez-Ruiz et al. 2014). It is hypothesised that the over-consumption of fat might change the sensitivity of receptors or the expression of receptors on the tongue, and might also change the physiological responses (such as secretion of satiety-related hormones), which could result in more stimuli (more fatty acids) being consumed and, hence, influence body weight change in the long-term (Batterham et al. 2002; Beglinger and Degen 2006; Breslin 2013; Stewart and Keast 2012). However, fat taste in most studies has been demonstrated by the gustatory sensation generated from fatty acids at detection levels (Chale-Rush et al. 2007a; Mattes 2009; Stewart et al. 2010; Stewart et al. 2011; Chevrot et al. 2014; Tucker et al. 2015), and there is little evidence to date linking this to the perception of fats in food. Therefore, the understanding of the association between fatty acid sensitivity and fat perception from dietary fat may help to understand underlying preference and choice of high fat foods. The influence of a high-fat diet on changing fatty acid sensitivity is reported by Stewart and Keast (2012) and Keast et al. (2014). In addition, subjects with varying sensitivity to fatty acid may show different physiological responses, which could further influence their fat intake (Keast et al. 2014). Therefore, understanding the impact of individual variation in fatty acid sensitivity on dietary intake could be important in reducing obesity.

Although several studies imply an association between fatty acid sensitivity, dietary intake and BMI, the studies generally have a small sample size with an under-representation of overweight/obese subjects and the results are conflicting (Chevrot et al. 2014; Martinez-Ruiz et al. 2014; Stewart et al. 2010; Stewart et al. 2011; Tucker et al. 2015). The research of Stewart et al. (2010) suggested that individuals with low sensitivity (hyposensitive, *n* = 42, 78 %) to fatty acids consumed significantly higher energy and fat and had significantly higher BMI in comparison to their hypersensitive counterparts (*n* = 12, 22 %). No overweight subjects were found in the hypersensitive group but there were 4 (9.5 %) in the hyposensitive group. A further study by Stewart et al. (2011) confirmed this finding in which the hyposensitivity group (*n* = 38, 74.5 %) contained 6 overweight and 1 obese subjects and, as before, no overweight/obese in the hypersensitivity group (where *n* = 13, 25.5 %). Such findings needed to be investigated in a larger study population in this study of 51 subjects, only 1 was obese. Interestingly, however, they noted that hyposensitive subjects consumed more full fat dairy and saturated fat and less low-fat products in comparison to the hypersensitive subjects. A larger study (*n* = 121) by Martinez-Ruiz et al. (2014) found that there was a negative association between fatty acid sensitivity and BMI (*p* = 0.03) and subjects who gave higher intensity ratings to linoleic acid (*n* = 30) had, on average, lower BMI (*p* = 0.04). Although there was a distribution of BMI within each rating group; the high-intensity rating group had only 7 (23.3 %) overweight/obese but 23 (76.7 %) underweight/normal subjects, whereas in the low-intensity rating group this was more evenly distributed at 13 (43.3 %) and 17 (56.7 %), respectively. However, this study did not find any relationship between different fat-intensity rating groups and their energy or nutrient intakes. The studies of Chevrot et al. (2014) and Tucker et al. (2015) failed to find the relationship between fatty acid sensitivity, BMI or body fat.

Therefore, the aims of this study are as follows:

1. To examine the effects of different sensory modalities (taste, mouthfeel and odour) on perceived intensity of fat in a real food model
2. To investigate the relationship between individual fatty acid sensitivity and oral fat perception in food
3. To explore the association between fatty acid sensitivity, BMI and fat intake

There are two sub-studies. The first study was conducted in order to examine the effects of sensory modalities on fat perception and to explore the association between fatty acid sensitivity, fat perception, BMI and dietary intake. This study was also regarded as a pilot study in order to calculate the sample size for the later study. Study 2 was designed to obtain a more comprehensive understanding of the relationship between fatty acid sensitivity and fat perception, through using more levels of fat but fewer modalities.

## Materials and Methods

### Study 1

#### Participants

Forty-six participants were recruited for study 1. The study was given approval by the University of Reading Research Ethics Committee (Ethics 12/04). All subjects were 18–55 years old and they were non-smokers with no allergies to dairy products. Those suffering from medical conditions affecting taste or smell of food, on a weight-reducing diet, using medication likely to affect taste, odour or appetite, with a history of alcohol or drug abuse/addition or breast-feeding or pregnant, were excluded from the study. They were asked not to eat, nor to drink strong beverages (such as coffee), for 1 h prior to the visit.

The study consisted of one visit where participants were asked to rate the intensity of different sets of milk/cream samples with or without nose clips. Weight and height, as well as fatty acid detection threshold and food intake data, were collected.

#### Sample Preparation for Fat Intensity Test

Non-fat skimmed milk, single cream (19.1 % fat content) and double cream (50.5 % fat content) (Tesco, UK) were used to produce five different fat level samples, 0, 7.5, 10, 15 and 20 %. Two sets of samples were prepared, mouthfeel-masked samples (MM) and mouthfeel-non-masked samples (MNM). For MM samples, an easy to disperse maltodextrin- and xanthan gum-based thickener (Nestlé Nutrition Resource ThickenUp Clear, Liverpool, UK) and liquid paraffin (Care, Thornton & Ross, Huddersfield, UK) were used to mask the viscosity and lubricity. The composition of each sample is shown in Table 1. The thickener and liquid paraffin were added after the combination of milk and cream had been blended. After mixing the components together, samples (100 ml) were homogenised at 5000 rpm for 3 min using a high-shear mixer (Silverson Laboratory Mixer, Silverson machines, Chesham, UK). The samples were prepared daily, 1 h prior to testing. The samples (15 ml) were served at ambient temperature (23 ± 2 °C) to each participant. The viscosity results of samples were measured by Bohlin CVO rotational rheometer, and the details are shown in Supplementary Online Resource 1. Although the particle size and emulsion stability were not measured in this study, the use of the high-shear homogeniser was to ensure a small and uniform particle size, and the thickener used (which was predominantly xanthan gum) had both emulsification and stabilisation properties (García-Ochoa et al. 2000; Katzbauer 1998).

**Table 1:** The compositions of mouthfeel-masked and mouthfeel-non-masked samples

Fat content (g/ml)	Non-fat skimmed milk (% *v*/*v*)	Single cream (% *v*/*v*)	Double cream (% *v*/*v*)	Thickener (% *w*/*v*)	Liquid paraffin (% *w*/*v*)
				These were only used in mouthfeel-masked (MM) samples	
0 %	100 %	0 %	0 %	1.60 %	5 %
7.5 %	60.5 %	39.5 %	0 %	1.25 %	5 %
10 %	60.5 %	31.7 %	7.8 %	1.20 %	5 %
15 %	60.5 %	15.8 %	23.7 %	1.10 %	5 %
20 %	60.5 %	0 %	39.5 %	1 %	5 %

#### Sample preparation for fatty acid sensitivity (detection threshold)

Food-grade oleic acid (Sigma, UK) was chosen, as it is an abundant long-chain monounsaturated fatty acid, which accounts for up to 20–30 % of total fatty acid in dairy products, such as milk, (Mansson 2008). The control sample consisted of 0.01 % *w*/*w* EDTA, 0.95 % *w*/*w* thickener, 2.86 % *w*/*w* liquid paraffin, 77.13 % *w*/*w* non-fat skimmed milk (Tesco), 19.05 % *w*/*w* deionised water. 0.01 % *w*/w EDTA was dissolved in deionised water (at 50 °C with agitation), and added to prevent the oxidation of oleic acid. The thickener used was Nestlé Nutrition Resource ThickenUp Clear, which easily dissolved in both water and milk and did not contribute to appearance nor flavour. Thickener and liquid paraffin were applied to mask the viscosity and lubricity. The concentration of oleic acid was from 0.31 to 31.4 mM (0.0088 to 0.89% *w*/*v*), with dilution differing by 0.25 log units. This range was informed by the studies of Chale-Rush et al. (2007a) and Mattes (2009); in addition, this range covered the detection threshold found by Stewart et al. (2010). The same homogenisation conditions used for the intensity samples were applied (100 ml samples homogenised for 3 min at 5000 rpm). Samples were prepared daily, 1 h prior to testing. The samples (20 ml) were served at ambient temperature (23 ± 2 °C).

#### Procedure for Rating Fat Intensity

Participants were asked to taste the milk/cream samples and rate the perceived “fattiness” intensity (fat intensity). Mouthfeel-masked and mouthfeel-non-masked samples were provided. Nose clips were provided for odour-masked samples. The generalised labelled magnitude scale (gLMS) was used for fat intensity rating, ranging from “no sensation” (0) to “strongest imaginable sensation of any kind” (100). This scale was modified from the labelled magnitude scale, which was reported to be better to compare the intensity results between different taste modalities and between sensitivity groups (Bartoshuk et al. 2004).

**Fig. 1:**
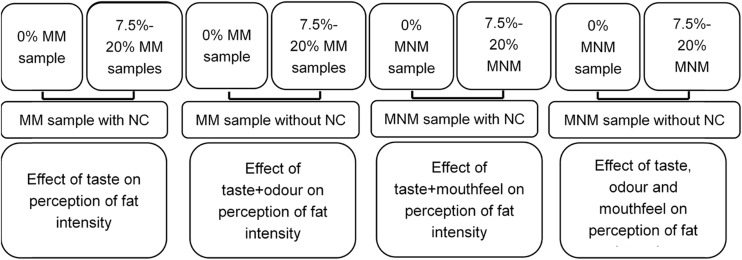
The procedure of fat intensity rating and the rationale for each set of samples. *MM* mouthfeel-masked, *MNM* mouthfeel-non-masked, *NC* nose clip

Recruited participants were firstly asked to rate the perceived fat intensity of mouthfeel-masked samples with nose clips. The 0 % fat sample was first served to participants to rate the intensity. This sample was labelled as “reference” and described to the participants as a reference sample which contained 0 % fat. Subsequently samples of different fat contents (from 7.5 to 20 %), labelled with three-digit codes, were presented randomly in a balanced order. Participants were then asked to remove the nose-clips. They then rated the mouthfeel-masked samples again followed by another 0 % fat sample (labelled as “Reference”). The samples were presented with different codes and in a randomised balanced order. Mouthfeel-non-masked samples labelled with three-digit random codes were then assessed using the same procedure, first with nose clips and then without nose clips. Figure 1 illustrates the procedure of fat intensity rating, as well as the rationale behind each set of samples.

The testing was conducted under red light to mask any visual difference between samples. Warm filtered tap water (approximately 40 °C) and low-salt and low-fat (less than 1 %) crackers (Carr’s Table Water crackers, United Biscuits, Hayes, UK) were provided to help participants to cleanse their mouth. There was a 40-s time delay between samples and participants were instructed to rinse their mouth using the water and crackers.

#### Procedure for determining fatty acid sensitivity (detection threshold)

The rapid 3 alternative forced choice (3-AFC) approach developed by Allen et al. (2014) was modified to measure fatty acid detection thresholds in this study, in order to avoid assessor fatigue, especially when wearing nose clips. Participants were provided crackers and warm water to rinse their mouth. They were asked to expectorate the sample into the provided spittoon after each tasting. During the whole process, participants were asked to wear nose clips all of the time to avoid any olfactory effects.

**Fig. 2:**
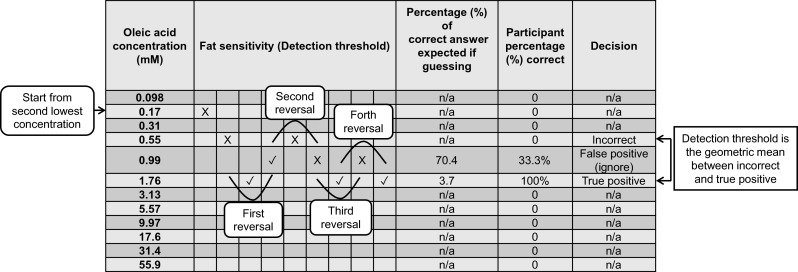
The procedure of sample presentation to determine an individual’s detection threshold, which is modified from the rapid ascending 3-AFC method developed by Allen et al. (2014). This figure here is an example based on the result of one participant. The percentage (%) correct if guessing was calculated based on binomial expansion. The participant’s actual percentage (%) correct was calculated as the ratio of the number of correct answers to the total number of sample sets presented to the participant at the corresponding fatty acid level (Formulas are shown in Supplementary Online Resource 2)

Three samples were provided in a set, one fatty acid sample and two controls, and participants were asked to select the odd one out. In the first sample set, the second lowest concentration of oleic acid was presented (0.55 mM). If the incorrect sample was chosen, the next set of samples would be a higher concentration of oleic acid by two levels until the participant identified the correct sample in a set, which achieved the first reversal. After a correct identification, a lower concentration of oleic acid was given by one level (Fig. 2). The procedure continued until participants repeatedly selected the correct sample at one concentration following a minimum of four reversals.

To determine an individual’s detection threshold, the participant percentage (%) correct at each level presented was calculated, which was the ratio of the correct answers to the total number of sample sets served at this fatty acid level. If the percentage correct was significantly greater than expected by chance (percentage (%) correct if completely guessing), the answer was regarded a true positive. If less than expected by chance (percentage (%) correct if completely guessing), it was regarded as false positive and was ignored. The detection threshold was calculated as the geometric mean between the lowest concentration that was a true positive and the concentration considered as the highest incorrect answer.

#### Anthropometric and Dietary Intake Measurements

Height was measured by a wall-mounted stadiometer and weight was measured on a glass electronic balance (Salter, UK). BMI was calculated by the Quetelet Index (kg/m^2^).

A food frequency questionnaire (EPIC-Norfolk, UK) was used to obtain the nutrient intakes of participants. It was analysed by FETA software (v2.53, UK).

### Study 2

Several details, such as the fat levels of samples used in intensity rating and the number of participants, were modified for study 2 based on the results of study 1.

#### Participants

Sample size calculation with alpha risk at 0.05 and beta risk at 0.2 (power 80 %) was performed based on the intensity rating results of study 1. With the aim of finding a significant difference between fat levels, it was estimated that 50 participants were required.

Fifty-one participants were recruited in study 2. The modified study ethics was given approval by the University of Reading Research Ethics Committee. The criteria for recruiting participants were the same as those in study 1 (2.1.1); in addition, those who participated in study 1 were excluded.

Each participant attended two visits in study 2. During the first visit, participants were asked to rate perceived fat intensity of the different sets of milk/cream samples with or without nose clips. Detection threshold, weight and height were collected in this visit. Participants were then given the Food Frequency Questionnaire (developed and validated by European Prospective Investigation into Cancer and Nutrition (EPIC) group, Riboli et al. 2002) to complete and return at their second visit. During the second visit participants undertook 2-AFC tests on a series of milk/cream samples with varying fat levels.

#### Sample Preparation for Fat Intensity Test

As commercial fat levels in dairy beverages are from 0 to 10 % in the UK market, and fat perception was considered to reach a continuum plateau at 15 to 20 % fat level based on intensity results in study 1, additional fat levels of 2.5 and 5 % were added into study 2 to more accurately reflect the market place.

Seven fat level samples, 0, 2.5, 5, 7.5, 10, 15 and 20 %, were prepared using skimmed milk, single cream, double cream (Tesco, UK) and water. Two sets of samples were presented, mouthfeel-masked (MM) samples and mouthfeel-non-masked (MNM) samples. Thickener and liquid paraffin were used to mask viscosity and lubricity as in study 1. The water was added in order to keep the sugar level constant (3.66 % g/ml) in each sample. The composition of each set of samples is shown in Table 2. After mixing the components together, samples (100 ml) were homogenised at 5000 rpm for 3 min using the high-shear mixer. The mixed samples were prepared daily, 1 h prior to testing, and served (20 ml) at ambient temperature (23 ± 2 °C).

**Table 2:** The composition of mouthfeel-masked (MM) and mouthfeel-non-masked (MNM) samples used to rate fat intensity

Fat content (g/ml)	Non-fat skimmed milk (% *v*/*v*)	Water (% *v*/*v*)	Single cream (% *v*/*v*)	Double cream (% *v*/*v*)	Thickener (% *w*/*v*)	Liquid paraffin (% *w*/*v*)
					These were only used in mouthfeel-masked (MM) samples	
0 %	73 %	27 %	0	0	1.60 %	5 %
2.5 %	67.7 %	19.6 %	12.7 %	0 %	1.50 %	5 %
5 %	61.8 %	12.4 %	25.8 %	0 %	1.35 %	5 %
7.5 %	55.8 %	4.7 %	39.5 %	0 %	1.25 %	5 %
10 %	56.8 %	3.7 %	31.7 %	7.8 %	1.20 %	5 %
15 %	58.7 %	1.8 %	15.8 %	23.7 %	1.10 %	5 %
20 %	60.5 %	0 %	0 %	39.5 %	1 %	5 %

#### Procedure for Rating Fat Intensity

Participants were asked to rate the fat intensity of both mouthfeel-masked and mouthfeel-non-masked samples. Nose clips were worn with mouthfeel-masked samples in order to mask odour.

**Fig. 3:**
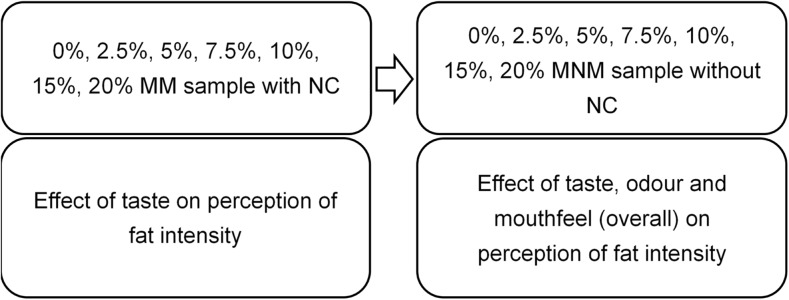
The procedure of fat intensity rating in study 2 and the rationale behind each set of samples; *MM* mouthfeel-masked, *MNM* mouthfeel-non-masked, *NC* nose clip

The extent of influence of mouthfeel and odour on fat perception was established in study 1; therefore, in study 2, the primary aim was to determine whether participants could distinguish differences between fat levels. The secondary aim was to determine any relationship between fatty acid sensitivity and perceived fat intensity in a real food model. In addition, fatty acid sensitivity might be expected to be more associated with perceived fat intensity from a real food model under the condition where mouthfeel and odour were both masked. Therefore, only “taste” (mouthfeel and odour both masked) and “overall” (no masking) modalities were tested. Participants were first asked to rate the perceived fat intensity of mouthfeel-masked samples with nose clips. The 0 % fat sample was initially given to participants to rate as a reference sample as in study 1. However, in study 2, the 0 % sample was also provided blind coded and in a randomised balanced order with the other samples. Therefore, seven different fat content samples (from 0 % to 20 %) with three-digit random codes were provided in a randomised balanced order to participants. Mouthfeel-non-masked samples were provided using the same procedure. Figure 3 illustrates the full procedure of fat intensity rating, as well as the rationale behind each set of samples. The testing was conducted under red light to mask any visual difference between samples. There was a 40-s time delay between samples, and participants were instructed to rinse mouth by using warm water and crackers.

#### Procedure for comparing fat intensity (two-alternative forced choice test)

The 2-AFC test was used in study 2 to ensure that if differences between samples were small this was not missed, as can happen when rating multiple samples presented monadically. The five milk/cream samples with 0 to 10 % fat levels described in Table 4 were used in this test. Participants were provided with two samples (with all the possible combinations of these five samples, ten sets of samples in total) each time and asked to choose the “fattier” sample. All sets were presented in a randomised balanced order. In mouthfeel-masked samples, participants were asked to wear the nose clips. The testing was conducted under red light to mask any visual difference between samples. There was a 40 s time delay between each set of samples. Warm water and crackers were provided for palate cleansing.

#### Sample preparation and procedure for determining fatty acid sensitivity (detection threshold)

Samples were made and tested using the same procedure described in study 1. Two lower concentrations of fatty acid samples were included, as it was found in study 1 that several participants could detect the lowest level (0.31 mM) sample. Moreover, one higher concentration sample was included in order to ensure that values for participants with higher thresholds were more accurate. Therefore, the range of oleic acid in study 2 was from 0.098 to 55.9 mM (0.0028 to 1.58% *w*/*v*) with 0.25 log unit dilution.

### Statistical Analysis

For both study 1 and study 2, fat intensity rating results were collected by Compusense at-hand (Compusense, Canada). Data were analysed by SPSS Statistics (IBM, version 22). Demographic data, intensity data and dietary intake data are presented as means ± standard error of mean (SEM). Mauchly’s test was applied before ANOVA analysis, in order to examine the assumption of sphericity for main effects of fat level, modality condition, and the interaction effect of fat level and modality. If the sphericity was violated, degree of freedom was corrected by using Greenhouse-Geisser estimates of sphericity. Two-way repeated measure ANOVA and pairwise comparisons with Bonferroni were used to evaluate differences in intensity ratings between fat levels and modality conditions. Mixed factorial ANOVA with Bonferroni pairwise comparison was used to evaluate the difference in intensity ratings between fat levels, modality conditions and three fatty acid sensitivity groups. V power programme (written by Virginie Jesionka and based on the Discrimination Test Planning and Analysis Tools developed by Tom Carr (Carr Consulting, 1215 Washington Ave. Suite 203 Wilmette, IL 60091 USA) was applied to analyse the data obtained from 2-AFC tests. Both Binomial expansion (guessing model) and Thurstonian model were used to determine the *p* value, power and d’ value. Significance level (*p* value) was 0.05, 2-tailed.

## Results

### Results of Study 1

#### Results of Demographics, Energy and Fat Intakes

There were 46 participants in study 1, 21 female and 25 male. The age range was from 19 to 53 years old, and BMI range was from 16.5 to 43.5 kg/m^2^ (Table 3).

**Table 3:** Demographic information, reported daily energy and fat intakes of participants in study 1. Values are expressed as mean ± SEM

		Number of participants	Proportion of participant	Age	BMI (kg/m2)	Range of BMI (kg/m2)	Energy intake (kCal)	Total fat intake (%total energy)	SFA intake (%total energy)	MUFA intake (%total energy)	PUFA intake (%total energy)
Gender	Female	21	46 %	21–53	23.2 ± 1.4	16.5–43.5	1925 ± 222	34.9 ± 1.0	12.6 ± 0.6	13.0 ± 0.5	6.2 ± 0.4
	Male	25	54 %	19–46	24.0 ± 0.7	17.6–32.2	2173 ± 374	35.3 ± 1.2	13.3 ± 0.6	13.3 ± 0.5	5.6 ± 0.4
Sensitivity	High	16	35 %	19–47	23.6 ± 1.8	16.5–43.5	2120 ± 270	36.7 ± 0.9	13.6 ± 0.6	13.8 ± 0.4	5.9 ± 0.3
	Medium	9	20 %	20–53	24.1 ± 1.2	19.0–30.5	2706 ± 1002	34.9 ± 2.2	12.7 ± 1.0	12.6 ± 0.8	6.6 ± 0.9
	Low	21	46 %	19–46	23.5 ± 0.8	17.5–32.7	1738 ± 150	34.0 ± 1.3	12.7 ± 0.7	12.8 ± 0.6	5.5 ± 0.4

*BMI* body mass index, *SFA* saturated fatty acid, *MUFA* monounsaturated fatty acid, *PUFA* polyunsaturated fatty acid

#### Results of Relative Effects of Modalities on Fat Intensity Rating

There was a significant main effect of fat level on intensity rating (F(3.29, 297.8) = 16.22, *p* < 0.0001). However, this was observed within “taste + odour” (*p* = 0.026), “taste + mouthfeel” (*p* < 0.0001) and overall modality (*p* < 0.0001).

However, the significant difference was only observed between 0 % and other fat level samples under taste + mouthfeel and overall conditions (*p* < 0.0001, *p* < 0.0001, respectively).

**Fig. 4:**
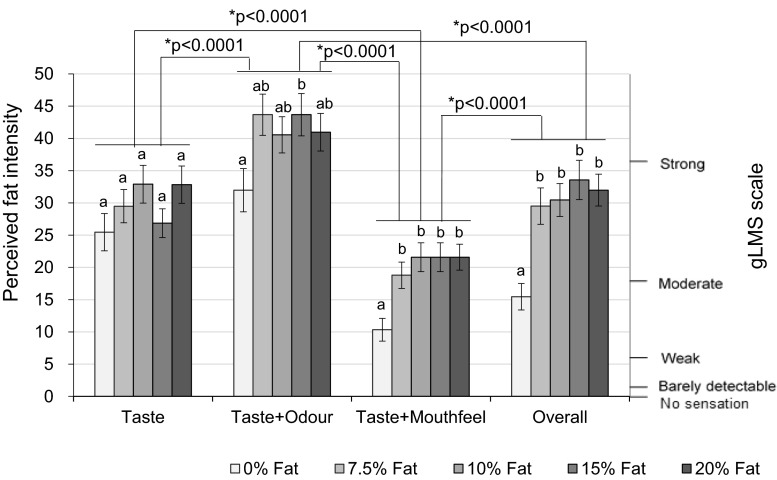
Perceived fat intensity under different modalities. *Bars* not sharing a common *letter* differ significantly (*p* < 0.05) between fat levels in one modality condition

Significant effects of modality on intensity rating was also observed (F(1.96, 977.29) = 27.59, *p* < 0.0001). Figure 4 illustrates the results of perceived fat intensity rated under different modality conditions for the five different samples.

Perceived fat intensity rated from taste + odour was significantly higher than the perceived intensity from taste (*p* < 0.0001). Similarly, the intensity from all modalities (overall) was rated higher than from taste + mouthfeel (*p* < 0.0001). This implies that odour contributes significantly to the perceived fat intensity.

Perceived fat intensity rated from taste + odour was significantly higher than overall (*p* < 0.0001); in addition, the intensity from taste was significantly higher than taste + mouthfeel (*p* < 0.0001). As the samples for taste + odour and taste contained liquid paraffin and thickener which increase the viscosity and lubricity, it is the increase in these mouthfeel parameters that has enhanced the perceived fat intensity.

#### Results of fatty acid sensitivity (detection threshold)

As the number of participants in our study is relatively small, the grouping results by quartile analysis could shift substantially depending on the individuals recruited. Therefore, the approach taken to categorise participants into different sensitivity groups, was to divide the logarithmic scale into equal parts.

**Fig. 5:**
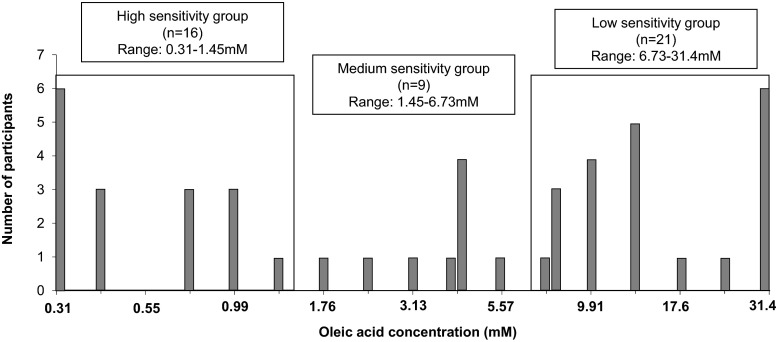
Oleic acid detection thresholds in milk of 46 participants in study 1

Those with detection thresholds above 6.73 mM were assigned to the low-sensitivity group and those with detection thresholds below 1.45 mM were assigned to the high-sensitivity group. Those in the middle range were assigned to the medium-sensitivity group (Fig. 5). There were 16 participants (35 %) in high-sensitivity group (0.31 to 1.45 mM oleic acid, 0.0088 to 0.041 % *w*/*v*), 9 (20 %) in medium sensitivity group (1.45 to 6.73 mM oleic acid, 0.041 to 0.19% *w*/*v*) and 21 participants (45 %) within the low-sensitivity group (6.73 to 31.4 mM oleic acid, 0.19 to 0.89% *w*/*v*).

#### Relationship between Fatty Acid Sensitivity and Fat Perception

In the milk/cream mixture, there was no significant effect of sensitivity group on the perceived fat intensity (F(2, 104.67) = 0.058, *p* = 0.944). In addition, there was no significant interaction between sensitivity groups, fat levels or modality conditions on the perceived fat intensity rating (F(15.92, 328.77) = 0.62, *p* = 0.86).

For both low and medium fatty acid sensitivity groups, a significant difference in perceived fat intensity rating was only observed between 0 % and the other fat level samples under the overall condition (in low-sensitivity group, 0 and 7.5 %: *p* = 0.004, 0 and 10 %: *p* = 0.004, 0 and 15 %: *p* < 0.0001, 0 and 20 %: *p* = 0.004; in medium-sensitivity group, 0 and 7.5 %: *p* = 0.022, 0 and 20 %: *p* = 0.005). Whereas, there was no significant difference in perceived fat intensity between fat levels under any of the other three modality conditions.

**Fig. 6:**
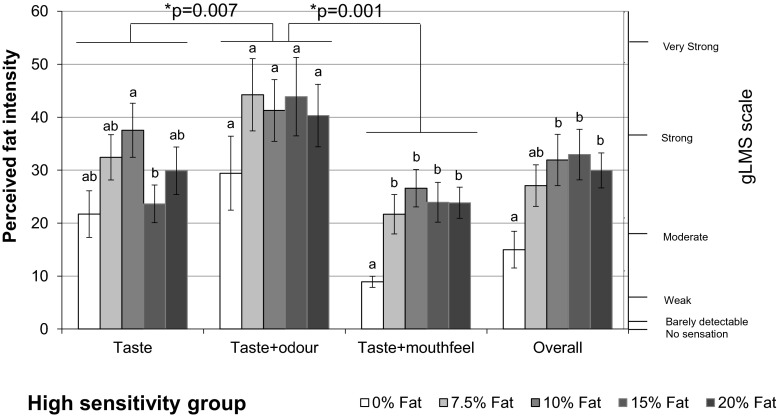
Perceived fat intensity under different sensory modalities for the high fatty acid sensitivity group. *Bars* not sharing a common *letter* differ significantly (*p* < 0.05) between fat levels within each modality condition

For the high-sensitivity group, significant differences were found in rating perceived fat intensity between 0 and 10 % (*p* = 0.001), 15 % (*p* = 0.007) and 20 % (*p* = 0.018) fat samples under the “Overall” modality condition (Fig. 6). In addition, there was a significant difference found between the 10 and 15 % fat samples under the “Taste” modality in this group (*p* = 0.008). Moreover, significant differences were found between 0 % and the other fat levels under the “Taste + Mouthfeel” modality (0 and 7.5 %: *p* = 0.004, 0 % and 10 %: *p* < 0.0001, 0 % and 15 %: *p* = 0.001, 0 % and 20 %: *p* = 0.001).

#### Relationship between fatty acid sensitivity and BMI, energy and fat intake

No significant difference was found in BMI between the three fatty acid sensitivity groups (shown in Table 3). In addition, there was no significant difference in energy intake or fat intake between these different sensitivity groups.

### Results of Study 2

#### Results of demographics, energy and fat intakes

There were 51 participants in Study 2, 69 % female and 31 % male (Table 4). The age range was from 18 to 55 years old and BMI range was from 17.0 to 39.3 kg/m^2^.

**Table 4:** Demographic information, reported daily energy and fat intakes of participants in study 2. Values are expressed as mean ± SEM

		Number of participants	Proportion of participant	Age	BMI (kg/m2)	Range of BMI (kg/m2)	Energy intake (kCal)	Total fat intake (%total energy)	SFA intake (%total energy)	MUFA intake (%total energy)	PUFA intake (%total energy)
Gender	Female	35	69 %	18–55	23.5 ± 0.8	17.5–37.9	1918 ± 156	33.4 ± 1.2	12.2 ± 0.6	12.2 ± 0.5	5.8 ± 0.3
	Male	16	31 %	18–55	25.1 ± 1.5	18.2–39.3	1967 ± 168	33.8 ± 1.3	13.0 ± 0.7	12.2 ± 0.6	5.4 ± 0.4
Sensitivity	High	24	47 %	20–55	24.0 ± 1.1	18.0–39.3	2022 ± 213	34.1 ± 1.1	13.0 ± 0.6	12.5 ± 0.5	5.4 ± 0.3
	Medium	14	27 %	18–55	24.2 ± 1.2	17.6–32.3	1943 ± 153	33.9 ± 1.2	12.1 ± 0.6	12.8 ± 0.6	5.8 ± 0.4
	Low	13	25 %	18–51	23.8 ± 1.3	17.5–33.5	1758 ± 196	32.1 ± 2.6	11.7 ± 1.2	11.4 ± 1.0	6.0 ± 0.6

BMI body mass index, SFA saturated fatty acid, MUFA monounsaturated fatty acid, PUFA polyunsaturated fatty acid

#### Fat intensity ratings from “taste” and “overall” modalities

There was a significant effect of fat level on intensity ratings ( F(4.12, 206.14) = 31.37, *p* < 0.0001), which demonstrated that increased fat level increased perceived fat intensity. Significant difference was observed within taste (*p* = 0.001) and “overall” (*p* < 0.0001).

In addition, a significant effect of modality on perceived fat intensity was also found (F(1, 50) = 16.71, *p* < 0.0001), where the perceived fat intensity rated during taste modality was rated significantly higher than overall modality. The main reason causing this phenomenon was due to the addition of paraffin and thickener in the samples used for evaluating the effect of “Taste” modality, as discussed in study 1. A significant interaction of fat level by modality on perceived fat intensity rating was also observed (F(4.30, 214.92) = 3.19, *p* = 0.004).

**Fig. 7:**
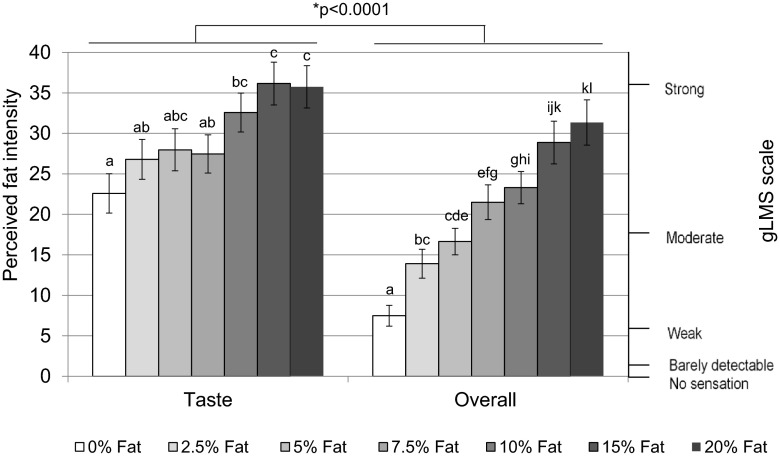
Perceived fat intensity under different modalities. *Bars* not sharing a common *letter* differ significantly (*p* < 0.05) between fat levels within one modality condition

Figure 7 presents the perceived fat intensity under the two tested modalities. For the “taste” modality, significant difference in perceived fat intensity rating were observed between 0 % (presented blind and balanced in set) and 10 % (*p* = 0.005), 15 % (*p* < 0.0001) and 20 % (*p* < 0.0001) fat content. In addition, intensity ratings were also significantly different between 2.5 and 15 % (*p* = 0.021), 20 % (*p* = 0.023), as well as between 7.5 and 15 % (*p* = 0.007), 7.5 and 20 % (*p* = 0.015) fat content.

Under the overall (all modalities) condition, the 0 % fat samples differed significantly from all other samples (2.5 %: *p* = 0.004; 5 %: *p* < 0.0001; 7.5 %: *p* < 0.0001; 10 %: *p* < 0.0001; 15 %: *p* < 0.0001; 20 %: *p* < 0.0001). Significant differences were also observed between many other sample pairs (as shown in Fig. 7), except between adjacent fat levels.

#### Discrimination between Fat Levels Identified Using the 2-AFC Test

Table 5 presents the results of the 2-AFC test. In the “taste” condition, the 0 % fat sample differed significantly from the other four samples; in addition, the 5 % sample was significantly different from the 7.5 and 10 % fat samples. The 2.5 % fat sample was significantly different from the 7.5 % sample. Although the 2.5 % fat sample was not found to differ significantly from the 5 and 10 % fat samples, and the distances between these sample pairs was small (d’ values of 0.32 and 0.40, respectively), these samples cannot be claimed to reach similarity in this study as the power was insufficient (35 and 46 %, respectively).

**Table 5:** Discrimination tests results for samples varying in fat contents, tested in pairs using the 2-AFC test. % = proportion of people that distinguished products

		Taste					Overall			
	Fat level	0 %	2.5 %	5 %	7.5 %	Fat level	0 %	2.5 %	5 %	7.5 %
Correct answers	2.5 %	33	–	–	–	2.5 %	42	–	–	–
%		29 %	–	–	–		66 %	–	–	–
*P* value		0.02	–	–	–		<0.0001	–	–	–
Power		66 %	–	–	–		100 %	–	–	–
d’ value		0.53	–	–	–		1.35	–	–	–
Correct answers	5 %	40	30	–	–	5 %	47	41	–	–
%		57 %	18 %	–	–		84 %	61 %	–	–
*p* value		<0.0001	0.13	–	–		<0.0001	<0.0001	–	–
Power		100 %	35 %	–	–		100 %	100 %	–	–
d’ value		1.12	0.32	–	–		1.99	1.22	–	–
Correct answers	7.5 %	39	40	33	–	7.5 %	50	49	42	–
%		53 %	54 %	32 %	–		96 %	88 %	68 %	–
*p* value		<0.0001	<0.0001	0.02	–		<0.0001	<0.0001	<0.0001	–
Power		99 %	99 %	68 %	–		100 %	100 %	100 %	–
d’ value		1.02	1.05	0.58	–		2.9	2.2	1.41	–
Correct answers	10 %	39	31	33	29	10 %	48	43	42	27
%		53 %	22 %	29 %	14 %		88 %	69 %	65 %	6 %
*p* value		<0.0001	0.08	0.02	0.2		<0.0001	<0.0001	<0.0001	0.39
Power		99 %	46 %	66 %	25 %		100 %	100 %	100 %	10 %
d’ value		1.02	0.4	0.53	0.25		2.2	1.44	1.32	0.11

In the “overall” condition, significant differences were found between most pairs of samples, except there was no significant difference between the 7.5 and 10 % fat samples d’ = 0.11, (insufficient power to claim similarity).

#### Results of fatty acid sensitivity (detection threshold)

**Fig. 8:**
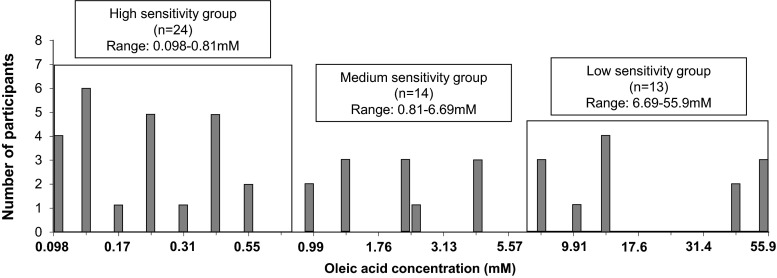
Oleic acid detection thresholds of 51 participants in Study 2

The approach to group participants was described in 3.1.3. Participants were divided into three sensitivity groups using the same approach as in section 3.1.3. There were 24 participants (47 %) in the high-sensitivity group (0.098–0.81 mM, 0.0028–0.023 % *w*/*v*), 14 (27 %) in the medium-sensitivity group (0.81–6.69 mM, 0.023–0.19 % *w*/*v*) and 13 (25 %) in the low-sensitivity group (6.69–55.9 mM, 0.19–1.58 % *w*/*v*) (Fig. 8).

#### Relationship between Fatty Acid Sensitivity and Fat Perception

No significant difference in the perceived fat intensity rating was found between fatty acid sensitivity groups (F(2, 1508.03) = 1.91, *p* = 0.342). There was no significant interaction between sensitivity groups, fat levels or modality conditions on rating (F(8.70,208.82) = 1.4, *p* = 0.193).

**Fig. 9:**
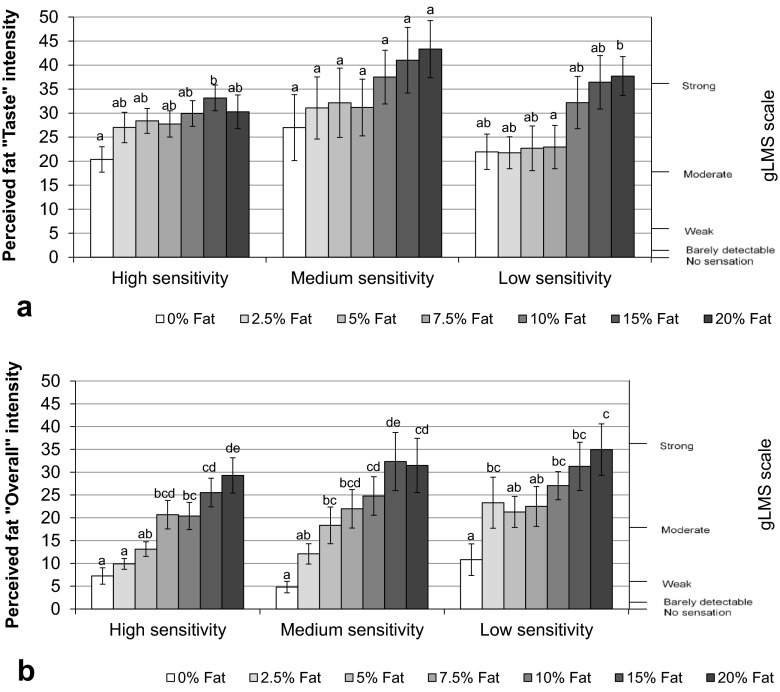
Perceived fat intensity under “Taste” modality (**a**) and under “Overall” modality (**b**) for three sensitivity groups. *Bars* not sharing a common *letter* differ significantly (*p* < 0.05) between fat levels within one sensitivity group

The low-sensitivity group showed a significantly different intensity rating between 7.5 and 20 % (*p* = 0.03) under “Taste” condition, whereas no significant difference was found between fat levels in the medium-sensitivity group (Fig. 9a). In the high-sensitivity group, significant difference was only observed between 0 and 15 % (*p* = 0.042).

There were more significant differences observed under the “Overall” condition in comparison to the “Taste” condition. All three sensitivity groups could detect the difference between 0 and 10 %, 15 %, 20 % fat content (*p* < 0.05) (Fig. 9b). The high-sensitivity group also could detect the difference between 2.5 and 7.5 % (*p* = 0.006), 10 % (*p* = 0.014), 15 % (*p* = 0.001), 20 % (*p* < 0.0001), between 5 and 15 % (*p* = 0.002), 20 %, and also between 10 and 20 % (*p* = 0.036) fat content.

#### Relationship between fatty acid sensitivity and BMI, energy and fat intake

No significant differences were found in BMI between the three fatty acid sensitivity groups (Table 4). In addition, there was no significant difference in energy intake or fat intake between the different sensitivity groups.

## Discussion

### The Effects of three Sensory Modalities on Perceived Fat Intensity

Fat has been proposed as the sixth taste sensation both in animals and humans. Recent work has suggested that CD36 (Gaillard et al. 2008; Laugerette et al. 2005; Ozdener et al. 2014; Zhang et al. 2011), G-protein coupled receptors (Abdoul-Azize et al. 2014; Briscoe et al. 2003; Martin et al. 2011) and some transduction pathways, such as delayed rectifying potassium (DRK) channel (Gilbertson and Khan 2014; Liu et al. 2005) and the transient receptor potential type M5 (TRPM5) channels (Kaske et al. 2007; Liu et al. 2011; Minaya 2014), could be responsible for fat taste. Fatty acids are reported to be the effective stimuli in fat taste (Gilbertson et al. 1997; Gilbertson et al. 2005; Tsuruta et al. 1999). However, due to the minor presence of fatty acids in normal foods, where most fat is consumed as triglyceride, a greater understanding of fat taste, and its relative importance, in real food systems is needed.

Our results infer that fat is perceived not only through mouthfeel and odour in a real food (milk and cream) model but also through taste. The perceived fat intensity of mouthfeel-masked samples was rated higher than in mouthfeel-non-masked samples. Mouthfeel-masked samples contained thickener and liquid paraffin which increased viscosity and lubricity, and this resulted in enhanced perception of fat intensity within this system. This confirms that mouthfeel, such as thickness and lubrication, is an important indicator in oral fat perception and can be augmented by non-lipid components, in this case added gum-based thickener and paraffin. As odour non-masked samples were rated higher than odour masked samples, this confirms that odour can enhance the perception of fat, which could be due to higher odour sensitivity in comparison to gustatory response in humans (Chale-Rush et al. 2007b).

Although study 1 did not show significant differences between fat levels under the “Taste” condition (*p* = 0.058), this was significant in study 2 (*p* = 0.001), which implies that fat can be perceived through the taste modality. However, ratings of fat intensity were not always significant between specific fat levels in either study 1 or 2 using the gLMS scale. When using the more sensitive forced choice discrimination test, more significant differences between fat levels were found. Overall, the results from study 2 demonstrated that the difference between samples caused by various fat levels could be detected by a taste cue (under the condition in which mouthfeel and odour were both masked), which implies that “taste” could be an element influencing the oral fat perception in food. Although the “taste” of fat is recently described as “oleogustus” by Running et al. (2015), this is still less recognised and less familiar in food for most of consumers.

Mouthfeel of fat, such as thickness, viscosity and lubricity, largely influences fat perception, however taste appears to be an additional important factor that affects fat perception in real food systems.

### The relationship between fatty acid sensitivity, perceived fat intensity in a food model, BMI, and nutrient intake

Fatty acids are reported to be the effective oral stimuli that stimulate receptors and transduction pathways, and activate the neuronal response, hence, triggering taste perception. Our results support the previous studies (Chale-Rush et al. 2007a; Mattes 2009; Running et al. 2015; Stewart et al. 2010; Stewart et al. 2011) in concluding that humans can sense fatty acids when texture and olfaction are both masked, and they are consistent with other studies finding individuals present different oleic acid detection thresholds (Chale-Rush et al. 2007a; Stewart et al. 2010).

As triglyceride is the major component in dietary fat and the amount of direct consumption of free fatty acids through dietary fat is small, it is important to understand the relationship between fatty acid sensitivity and oral fat perception in real foods. However, limited studies have focussed on this aspect. Our study did not find a significant relationship between detection sensitivity for oleic acid and differences in perceived fat intensity (under the “taste” and “overall” conditions) in the milk/cream food models in neither study 1 nor study 2. A previous study by Stewart et al. (2010) found that subjects with higher oleic acid sensitivity (lower thresholds) had a stronger ability to distinguish varying fat levels within custard samples with added oil, compared to low-sensitivity subjects, when they were asked to rank the fat levels in a “taste” only model. The rating scale used in our study has less power than the forced choice ranking test used in the study of Stewart et al. (2010). Although the present study demonstrated that low-sensitivity subjects rated fat intensity low and constant for all samples below 10 % fat, the difference between this and the higher sensitivity groups, which tended to increase their scores for fat intensity as fat level increased from 0 %, was not significant (Fig. 9a). This implies that a larger study, or a more sensitivity scaling technique, is needed to support these results.

Although we found no difference between sensitivity groups, the amount of free oleic acid in 0.1 and 3.5 % fat milk are reported to be very low at 2 and 25 mg/L, respectively, according to the research of Amer et al. (2013). Therefore, the oleic acid levels in the milk-emulsion samples with 0 and 2.5 % fat levels might be below the detection threshold level, as the lowest detection threshold was 0.098 mM (28 mg/L oleic acid, 0.0028% *w*/*v*). Considering the oleic acid range of the high-sensitivity group, oleic acid levels of the samples from 5 to 20 % fat might be slightly higher or around the detection threshold level of this group; however, for medium and low-sensitivity groups, free oleic acid levels in all the samples (from 0 to 20 % fat) might be lower than their detection thresholds., However, these two groups can still distinguish the fat levels when mouthfeel and odour cues are minimised. This implies that lingual lipase might play important role in hydrolysing triglycerides into free fatty acid and increasing the fatty acid level to help individuals sense the taste of fat, as proposed by several studies (Kulkarni and Mattes 2013; Stewart et al. 2010). This hypothesis could give support to the finding that humans can detect fat though the taste cue by the presence of fatty acids, and also could imply an underlying mechanism for the subject variations in fatty acid sensitivity through individual differences in lingual lipase as suggested by Kulkarni and Mattes (2013) and Voigt et al. (2014).

Several studies have reported that subjects with high sensitivity to fatty acids consume less energy and fat in their diet, and may have lower BMI, in comparison to low-sensitivity subjects (Stewart et al. 2010; Stewart et al. 2011). Similarly, Martinez-Ruiz et al. (2014) found a negative association between fatty acid sensitivity and both BMI. The study of Tucker et al. (2015) reported a negative association between total fat, monounsaturated fat intake and fat intensity rating results, but failed to find a relationship between intensity ratings and BMI (or body fat percentage). However, our results found no relationship between fatty acid sensitivity and BMI or nutrient intake. This discrepancy could be due to the different methodology for sensitivity measurements and dietary intake collection, to differences in study size (which varied from *n* = 46 in our study 1 or *n* = 51 in our study 2, to *n* = 51 in Stewart et al. (2011), *n* = 121 in Martinez-Ruiz et al. (2014)) or due to different proportions of overweight subjects in the studies (varying from 28 % in our study 1 and 27 % in study 2, to only 13 % in Stewart et al. (2011) and 32 % in Martinez-Ruiz et al. (2014)) which affects both the power and the reliability of the findings in each of these studies. However, regardless of limited sample size and differing methodologies used in studies to date, the hypothesis that fatty acid sensitivity could influence food consumption and body weight requires further justification. Although it is hypothesised that the overconsumption of fat might alter receptor sensitivity and change physiological response, which may then lead to the alteration of fat consumption and body weight in the long-term; change of body weight is a result of complex interactions of multiple factors. Therefore, it is important to understand the extent to which fatty acid sensitivity could influence food consumption and body weight.

### Limitations and Future Suggestions

There are some limitations that need to be considered in our study. In the study design, only one type of food model, the emulsion of milk and cream, was used to examine the relative effects of taste, mouthfeel and odour on fat perception. Further investigation of the relative importance of the three sensory modalities (mouthfeel odour and taste) on fat perception in other food matrices, such as solid food models, is needed.

In the fat intensity ratings, modality sample sets were tested in the same order for each participant (mouthfeel odour-masked first (taste), then mouthfeel-masked (taste + odour), odour masked (taste + mouthfeel) and no masking (overall)). As the taste of fat is considered to be less familiar to participants in comparison to the sensations caused by mouthfeel and odour it was a concern that participants would focus on mouthfeel and odour more easily than taste. Therefore, mouthfeel odour-masked (Taste) samples were examined first. In addition, the thickness of mouthfeel-masked samples was not masked by red light; two sets of samples varied in thickness whilst the other two sets did not. It was anticipated that participants would rate fattiness based upon thickness. Hence, the appearance of thickness could have biased the rating of fattiness within the constant thickness samples if they were rated amongst the thickness-variable samples.

In both fat intensity ratings and fatty acid sensitivity test, use of nose-clips cannot guarantee odour perception is eliminated. Some volatile compounds can stimulate both the activation of trigeminal nerves and odour receptors during orthonasal and retronasal olfaction (Dragich and Halpern 2008). However, trigeminal nerves are not only present in nose but also in oral cavity and recent studies suggested that fatty acids could trigger the activation of trigeminal neurons (Wajid and Halpern 2012; Yu et al. 2012), therefore, retro-nasal stimulation cannot be completely ruled out.

In the fatty acid sensitivity (detection threshold) test, only oleic acid was used. However, food is a complex system, even milk and cream contains many other fatty acids, such as palmitic acid, stearic acid and linoleic acid. The studies of Chale-Rush et al. (2007a) and Mattes (2009) both suggested that individuals have different sensitivities to different fatty acids. Therefore, it would be worthwhile to use other fatty acids or combinations of these fatty acids to measure the sensitivity of participants, in order to find the relationship between fatty acid sensitivity and fat perception in food models.

The results of our study are limited to the relatively small cohort size which was predominantly from a high socio-economic group and had a higher proportion of normal weight subjects compared to overweight and obese subjects. In order to extend the findings to the population the study should be extended, particularly to incorporate more overweight subjects.

Food frequency questionnaire (FFQ) is a retrospective method, which requires subjects to recall their current or past diet. It may cause underestimation or overestimation of dietary intake. It uses frequencies of food intake as an estimate of intake and, therefore, it is difficult to get a precise intake amount for each nutrient. Errors in memory result in the omission of foods from the questionnaire. In future studies, it may be of value to take a more accurate measure of both short and long term dietary intake as authors have shown that dietary fat intake itself can have a substantial effect on fatty acid thresholds (Keast 2016).

## Electronic supplementary material

Supplementary Online Resource 1 (DOCX 120 kb)

Supplementary Online Resource 2 (DOCX 15 kb)
