# Supplementary material for: Relative Effects of Sensory Modalities and Importance of Fatty Acid Sensitivity on Fat Perception in a Real Food Model
Source: Chemosens Percept. 2016 Jul 11;9:105–19. doi: 10.1007/s12078-016-9211-5 (PMC4989022; doi:10.1007/s12078-016-9211-5)
Supplement: Supplementary file 1 — (DOCX 120 kb) [file 12078_2016_9211_MOESM1_ESM.docx]

***Supplementary 1: Results of instrumental analysis of samples used in intensity ratings***

Viscosity of samples used in the intensity rating test was determined by Bohlin CVO rotational rheometer (Malvern, UK) with a 25mm diameter cone-plate measuring system. The setting parameters of the rheometer were modified based on previous research ([de Wijk et al. 2006](#_ENREF_1); [Prinz et al. 2006](#_ENREF_2)). The temperature was set to 25±2 °C, the shear rate was set from 0 to 150 s^-1^, the sweep time was 600s and the gap size was set at 150mm. Each sample was measured in duplicate. The results demonstrated that the viscosity of samples used in the intensity ratings, both in Study 1 and Study 2, were similar (Fig 1 and Fig 2).

***Fig 1*** *The viscosity of the samples prepared at five fat levels which were used in fat intensity rating of Study 1*

***Fig 2*** *The viscosity of the samples prepared at seven fat levels which were used in fat intensity rating of Study 2*

de Wijk RA, Prinz JF, Janssen AM (2006) Explaining perceived oral texture of starch-based custard desserts from standard and novel instrumental tests Food Hydrocolloids 20:24-34 doi:10.1016/j.foodhyd.2005.02.008

Prinz JF, Huntjens L, de Wijk RA (2006) Instrumental and sensory quantification of oral coatings retained after swallowing semi-solid foods Archives of oral biology 51:1071-1079 doi:10.1016/j.archoralbio.2006.05.008
