# Supplementary material for: Relative Effects of Sensory Modalities and Importance of Fatty Acid Sensitivity on Fat Perception in a Real Food Model
Source: Chemosens Percept. 2016 Jul 11;9:105–19. doi: 10.1007/s12078-016-9211-5 (PMC4989022; doi:10.1007/s12078-016-9211-5)
Supplement: Supplementary file 2 — (DOCX 15 kb) [file 12078_2016_9211_MOESM2_ESM.docx]

***Supplementary 2: Formula for calculating the participant percentage (%) correct and percentage (%) correct if guessing in fatty acid sensitivity test***

The percentage correct at each level is for each individual participant; it is the ratio of the number of correct answers to the total number of times that sample set has been presented to that participant. The equation is below:

$$Participant percentage \left( \% \right) correct= \frac{Number of correct answers}{Total number of occasions this sample set presented to participant} \times100\%$$

The percentage of correct answers expected if the participant was guessing is as calculated by the Binomial expansion :

$$The percentage \left( \% \right)of correct answer expected by guessing=\sum_{x=i}^{n} \frac{n!}{x_{i}!\left( n-x_{i} \right)!}p^{x_{i}}{(1-p)}^{n-x_{i}}$$

n: total number of sets of sample served to participant

x: number of the times that sample was correctly recognised

i: range from x to n (i=n-x)

p: guessing chance (the guessing chance of 3AFC is 1/3)
